# Supplementary material for: Metformin as an Adjuvant to Photodynamic Therapy in Resistant Basal Cell Carcinoma Cells
Source: Cancers (Basel). 2020 Mar 13;12(3):668. doi: 10.3390/cancers12030668 (PMC7139992; doi:10.3390/cancers12030668)
Supplement: Supplementary file 1 [file cancers-12-00668-s001.docx]

Supplementary Materials

Metformin as An Adjuvant to Photodynamic Therapy in Resistant Basal Cell Carcinoma Cells

Marta Mascaraque, Pablo Delgado-Wicke, Cristina Nuevo-Tapioles, Tamara Gracia-Cazaña, Edgar Abarca-Lachen, Salvador González, José M. Cuezva, Yolanda Gilaberte
and Ángeles Juarranz


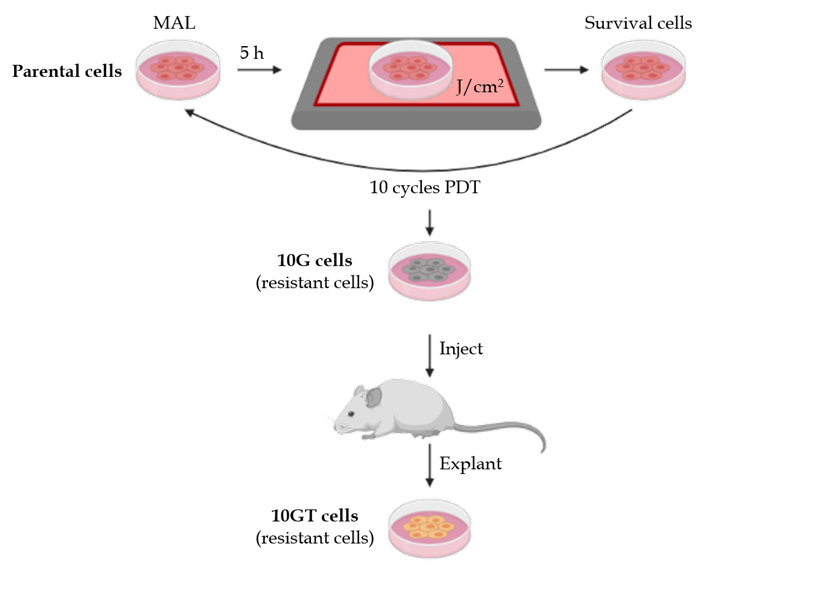


**Figure S1.** Schematic diagram of the protocol followed to generate both resistant cell lines.


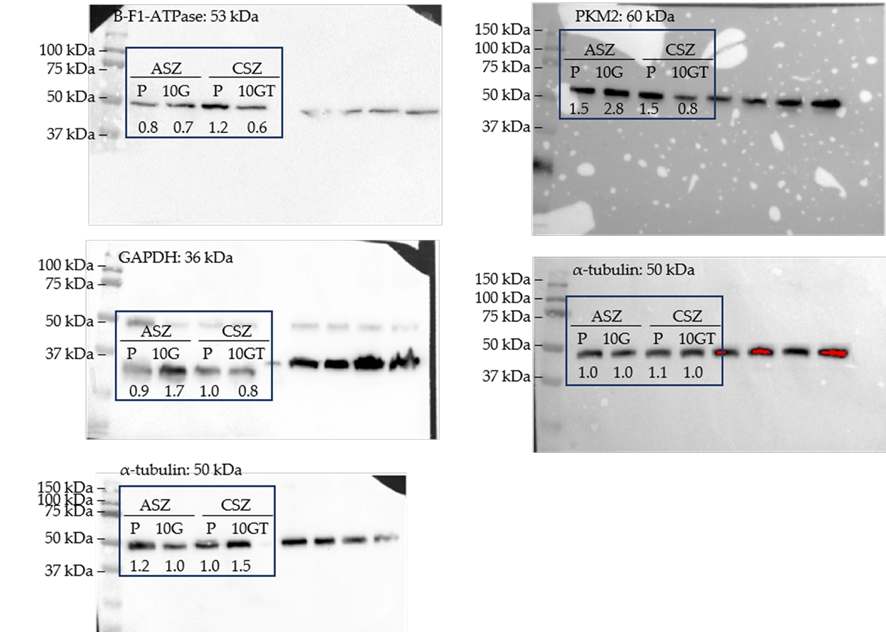


**Figure S2.** Full lenght immunoblot images from Figure 2b,c.


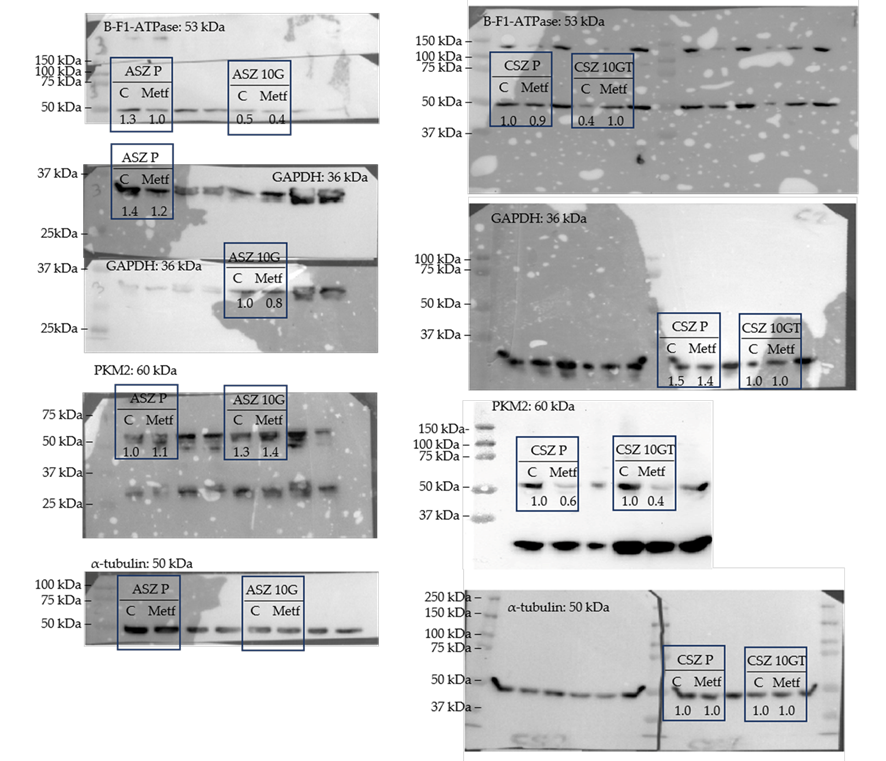


**Figure S3.** Complete Western blot images from Figure 3d,e.


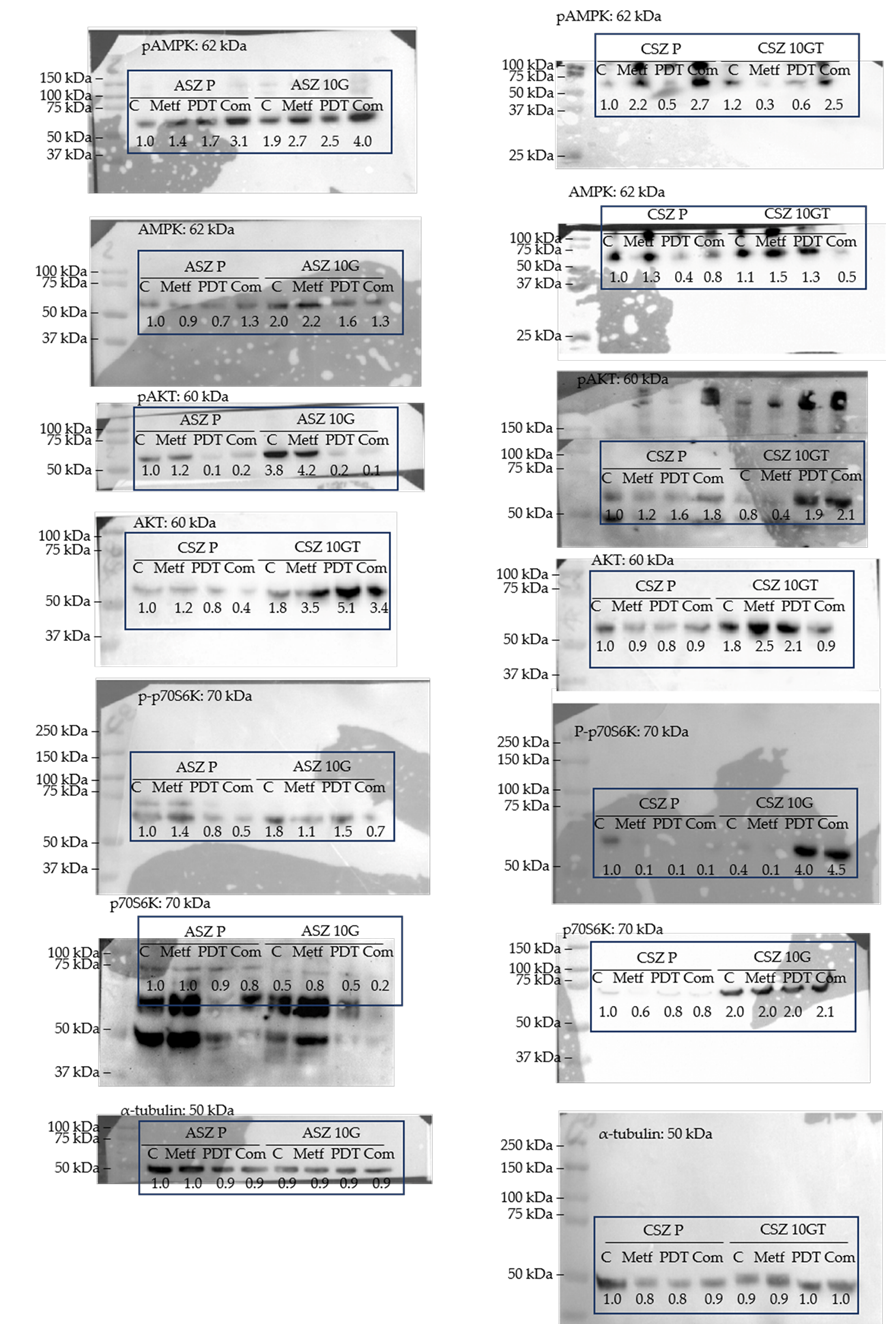


**Figure S4.** Full-length immunoblot images from Figure 5b.

**Table S1.** Summary of the metabolic changes observed in the resistant cells compared with its P cells.

|  | 10G ASZ vs. P ASZ | 10GT CSZ vs. P CSZ |
| --- | --- | --- |
| Proliferation | ns | ns |
| Β-F1-ATPase/ GAPDH |  |  |
| PKM2 |  | ns |
| OCR |  |  |
| OSR |  | ns |
| Lactate |  | ns |

**Table S2.** Summary of the metabolic changes observed after Metf treatment in all cell lines.

| Metf vs. Control | P ASZ | 10G ASZ | P CSZ | 10GT CSZ |
| --- | --- | --- | --- | --- |
| arrest G0/G1 phase |  |  |  |  |
| Β-F1-ATPase/ GAPDH | ns | ns | ns |  |
| PKM2 | ns | ns | ns |  |
| OCR |  |  |  | ns |
| OSR | ns |  |  | ns |
| Lactate |  |  |  |  |

| 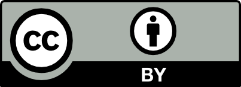 | © 2020 by the authors. Licensee MDPI, Basel, Switzerland. This article is an open access article distributed under the terms and conditions of the Creative Commons Attribution (CC BY) license (http://creativecommons.org/licenses/by/4.0/). |
| --- | --- |
